# New Mouse Model for Chronic Infections by Gram-Negative Bacteria Enabling the Study of Anti-Infective Efficacy and Host-Microbe Interactions

**DOI:** 10.1128/mBio.00140-17

**Published:** 2017-02-28

**Authors:** Daniel Pletzer, Sarah C. Mansour, Kelli Wuerth, Negin Rahanjam, Robert E. W. Hancock

**Affiliations:** Centre for Microbial Diseases and Immunity Research, Department of Microbiology and Immunology, University of British Columbia, Vancouver, Canada; Emory University School of Medicine

## Abstract

Only a few, relatively cumbersome animal models enable long-term Gram-negative bacterial infections that mimic human situations, where untreated infections can last for weeks. Here, we describe a simple murine cutaneous abscess model that enables chronic or progressive infections, depending on the subcutaneously injected bacterial strain. In this model, *Pseudomonas aeruginosa* cystic fibrosis epidemic isolate LESB58 caused localized high-density skin and soft tissue infections and necrotic skin lesions for up to 10 days but did not disseminate in either CD-1 or C57BL/6 mice. The model was adapted for use with four major Gram-negative nosocomial pathogens, *Acinetobacter baumannii*, *Klebsiella pneumoniae*, *Enterobacter cloacae*, and *Escherichia coli*. This model enabled noninvasive imaging and tracking of *lux*-tagged bacteria, the influx of activated neutrophils, and production of reactive oxygen-nitrogen species at the infection site. Screening antimicrobials against high-density infections showed that local but not intravenous administration of gentamicin, ciprofloxacin, and meropenem significantly but incompletely reduced bacterial counts and superficial tissue dermonecrosis. Bacterial RNA isolated from the abscess tissue revealed that *Pseudomonas* genes involved in iron uptake, toxin production, surface lipopolysaccharide regulation, adherence, and lipase production were highly upregulated whereas phenazine production and expression of global activator *gacA* were downregulated. The model was validated for studying virulence using mutants of more-virulent *P. aeruginosa* strain PA14. Thus, mutants defective in flagella or motility, type III secretion, or siderophore biosynthesis were noninvasive and suppressed dermal necrosis in mice, while a strain with a mutation in the *bfiS* gene encoding a sensor kinase showed enhanced invasiveness and mortality in mice compared to controls infected with wild-type *P. aeruginosa* PA14.

## IMPORTANCE

More than two-thirds of hospital infections are chronic or high-density biofilm infections and difficult to treat due to adaptive, multidrug resistance. Unfortunately, current models of chronic infection are technically challenging and difficult to track without sacrificing animals. Here we describe a model of chronic subcutaneous infection and abscess formation by medically important nosocomial Gram-negative pathogens that is simple and can be used for tracking infections by imaging, examining pathology and immune responses, testing antimicrobial treatments suitable for high-density bacterial infections, and studying virulence. We propose that this mouse model can be a game changer for modeling hard-to-treat Gram-negative bacterial chronic and skin infections.

## INTRODUCTION

The most threatening nosocomial infections are caused by the ESKAPE (*Enterococcus faecium*, *Staphylococcus aureus*, *Klebsiella pneumoniae*, *Acinetobacter baumannii*, *Pseudomonas aeruginosa*, and *Enterobacter* species) pathogens, which are increasingly demonstrating multidrug resistance to most or all available antimicrobial classes (1, 2). Indeed, in 2016, the United Nations called antimicrobial resistance a global risk and the largest threat to modern medicine, in part due to inadequate development of new antimicrobials. New animal models that can more rapidly assess the efficacy of novel antimicrobials against recalcitrant bacterial infections would contribute to much-needed drug development.

Early diagnosis of bacterial infections in an individual generally enables treatment with antibiotics. However, if the infection is not treated quickly or if treatment utilizes inappropriate antibiotics (e.g., due to resistance), it can lead to high-density or chronic infections or sepsis, which are conditions that are difficult to treat and that often have poor prognosis (3). While most acute infections are treatable with the current repertoire of antibiotics, chronic infections can persist for a long time by resisting multiple courses of antibiotic treatment and evading host defense mechanisms (4). To model the treatment of human infections, pathogenic bacteria have been extensively studied using acute infection mouse models (5). These models are not always ideal. For example, acute infections in various animal models by the Gram-negative bacterium *Pseudomonas aeruginosa* show great inconsistency and are rapidly lethal at higher infecting doses, while infections at lower infecting doses often resolve rapidly (6).

Other models are often highly invasive and ethically or technically challenging. For example, the burn-wound infection model is often used for Gram-negative infections. In this model, a wound is created on the back of a mouse or a rat by thermal injury of the skin with boiling water, ethanol (EtOH), flame, or a heating block, and bacterial suspensions are subsequently inoculated topically (7). For *Pseudomonas*, levels of bacterial inocula can range from low (10 organisms) to high (1 × 10^7^), but death of most animals occurs within less than a day. Alternatively, McRipley and Whitney (8) described a surgical site infection model in which a superficial incision was created on the back of each mouse prior to insertion of a suture inoculated with <10^5^ cells of *P. aeruginosa*. Although all mice developed clinical signs of infection, the results were not striking, and many mice showed rapid mortality even at low infection doses. These reflect many *P. aeruginosa* animal models that commonly demonstrate rapidly invasive short-term infections, technical challenges, and inconsistent data.

Only a few animal models have focused on longer-term chronic Gram-negative bacterial infections; of the available models, most require the organisms to be embedded in a biofilm-like matrix such as agar, alginate, or agarose (9) to prevent clearance by the host. Models that do not utilize embedded bacteria require a preformed biofilm on either a catheter or implant to induce a longer-term infection (10). Other limitations of these models involve reproducibility, sustainability, animal husbandry, and inaccurate mimicking of a human disease (11). Here we report on an alternative simpler model of chronic and progressive Gram-negative bacterial infection (including three Gram-negative ESKAPE pathogens as well as *Escherichia coli*) that enables the study of therapy, pathogenesis, and host responses. The model involves a subcutaneous method of delivery of bacteria that creates either a long-term abscess infection that causes local pathology or, by utilizing more-invasive strains, a shorter-term invasive and lethal infection enabling one to determine the role of various virulence determinants. The model overcomes many of the limitations mentioned above in that it requires no invasive animal procedures or manipulation/encapsulation of bacteria, is technically simple, is reliable and reproducible, and enables application of imaging technologies.

We demonstrate here that early local treatment of these high-density infections with antibiotics had a significant but incomplete impact on disease progression, reflecting the reality of antimicrobial therapy of infections containing high bacterial concentrations.

## RESULTS

### Identification of a *P. aeruginosa* strain able to cause a chronic cutaneous infection.

To initiate a cutaneous infection, we initially tested *P. aeruginosa* PA14.*lux*, a derivative of PA14, a well-known highly virulent clinical isolate (12), in a skin abrasion model with female CD-1 mice. Even at high inocula (10^7^ to 10^8^ CFU), infections were highly variable between individual mice but by day 3 luminescence decreased in 7 of 8 mice by 15- to 1,000-fold (see, e.g., Fig. S1a in the supplemental material). In an attempt to improve the consistency in this model, we induced neutropenia in CD-1 female mice by injecting cyclophosphamide 4 and 2 days prior to bacterial infection. Large bacterial doses of 10^6^ to 10^8^ CFU were rapidly lethal due to the loss of neutrophils, a major innate immune mechanism of *Pseudomonas* clearance. Even at the lower inocula of 10^3^ to 10^5^, 3 of 7 mice reached a humane endpoint by 48 h, 1 mouse failed to establish an infection, and 3 mice cleared infections by day 4 or 5 (see, e.g., Fig. S1b).

10.1128/mBio.00140-17.1 FIG S1  Abrasion model using the bioluminesently tagged strain *P. aeruginosa* PA14.lux in female CD-1 mice. The back of each mouse was shaved, and the skin was abraded using a nail file prior to application of bacteria in saline solution to the abraded area. The progress of infection was monitored daily using a PerkinElmer *in vivo* imaging system (IVIS). (a) Immunocompetent mice were infected with 10^7^ or 10^8^ CFU of *P. aeruginosa* PA14.lux. Three mice at an input dose of 10^8^, representative of 8 studied, are shown. All mice established an infection by day 1, but in 7 of 8 mice luminescence decreased substantially by day 2 and almost completely by day 3, while 1 mouse (on the right of panel a) established a chronic infection. Three further experiments were conducted that showed rather similar results to those of the two mice shown on the left hand panel of panel a. (b) Immunocompromised mice were made neutropenic by two treatments with 200 mg/kg cyclophosphamide 4 and 2 days prior to creating the abrasion. At bacterial input doses of 10^6^ and 10^8^ CFU, all mice reached their human-equivalent endpoint by day 2. At doses of 10^3^ and 10^5^ *P. aeruginosa* PA14.lux, 3 of 7 mice reached their lethal endpoint by day 2 (see, e.g., right-hand mouse in panel b), and the other 4 formed no infection or quite weak infections and were devoid of luminescent bacteria by days 4 to 6 (see, e.g., left-hand mouse in panel b). Download FIG S1, TIF file, 16.1 MB. Copyright © 2017 Pletzer et al. 2017 Pletzer et al. This content is distributed under the terms of the Creative Commons Attribution 4.0 International license.

Observing the inconsistency of the data obtained using the model described above led us to develop an alternative model where the endpoint of infection can be scored as a skin abscess in a quantitative manner. Initially, we injected *P. aeruginosa* PA14 underneath the thin skeletal muscle at the right dorsum on the back of shaved mice. At an input dose of 5 × 10^6^ organisms, all tested mice exhibited growth of *Pseudomonas* within a raised lump around the point of inoculum and formed skin abscesses above the raised lump. However, 40% of the 10 mice tested reached their humane endpoint by day 3 (Table 1), with mice dying at 24 h (1 mouse), 48 h (2 mice), and 72 h (1 mouse). A lower dose of 10^6^ organisms failed to induce abscess formation, while 10^8^ bacteria led to rapid lethality (i.e., humane endpoint within 16 h). Similar data were obtained with the well-defined, moderately virulent *P. aeruginosa* PAO1 laboratory strain. After various organs were harvested from surviving infected animals, it was found that both strains were able to disseminate throughout the body, heavily infiltrating the kidney and liver, although no bacteria were recovered from the spleen or lymph nodes. Since we assumed that dissemination of *Pseudomonas* from the infection site might be a limitation in establishing a chronic infection, we tested a *fliI* mutant of strain PA14. As hypothesized, it was able to persist locally, formed cutaneous lesions, and did not disseminate (Table 1).

**TABLE 1:** Influence of selected transposon mutants of *P. aeruginosa* PA14 on abscess infections

Strain	Product or function	Area of dermonecrosis (mean ± SE; mm^2^)	Geometric mean CFU per abscess	Mouse survival rate (%)	Infected kidney (%)	Infected liver (%)
*P. aeruginosa* PA14	Wild type	76 ± 15	1.23 × 10^8^	60	80	50
*P. aeruginosa fliI*	Flagella/motility	23 ± 13^a^	3.03 × 10^7^	100	0^b^	0
*P. aeruginosa wzz*	LPS O-antigen^e^	121 ± 92	1.49 × 10^8^	75	75	75
*P. aeruginosa fleQ*	Matrix PS regulation^f^	122 ± 70	2.13 × 10^8^	75	50	100
*P. aeruginosa rhlR*	Rhl quorum sensing	131 ± 41	1.42 × 10^7^^a^	100	50	25
*P. aeruginosa lasI*	Las quorum sensing	129 ± 31	3.34 × 10^8^	100	75	50
*P. aeruginosa* Δ*pchAD/*Δ*pvdGL*^c^	Pyochelin/pyoverdine	0^a^	1.63 × 10^3^^a^	100	0^b^	0
*P. aeruginosa lipA*	Lipase	141 ± 117	4.01 × 10^8^	50	100	100
*P. aeruginosa lasB*	Elastase	47 ± 23	3.29 × 10^7^	50	75	75
*P. aeruginosa toxA*	Exotoxin A	48 ± 43	2.47 × 10^8^	50	50	50
*P. aeruginosa exsA*	Type III secretion regulation	1 ± 1^a^	1.78 × 10^5^^a^	100	0^b^	0
*P. aeruginosa exoU*	Exoenzyme U	34 ± 17	1.31 × 10^7^	100	50	50
*P. aeruginosa phzA2*	Phenazine	75 ± 19	1.46 × 10^8^	75	75	75
*P. aeruginosa algR*	Global regulator	95 ± 22	1.47 × 10^8^	100	25	0
*P. aeruginosa bfiS*	Biofilm 2-component regulator	n.d.^d^	1.22 × 10^9^^d^	0	100	100
*P. aeruginosa phoP*	2-component response regulator of PhoPQ	53 ± 6	2.66 × 10^8^	75	50	50
*P. aeruginosa phoQ*	2-component sensor kinase	138 ± 52	2.07 × 10^8^	60	50	50

^a^ Significant difference from the wild type (*P* < 0.05; two-tailed Mann-Whitney test).

^b^ significant difference from the wild type (*P* < 0.05; two-tailed Fisher exact test).

^c^ PA14 pyochelin/pyoverdine deletion mutant.

^d^ Mice with very low survival. The data reflect only surviving animals, and no statistics could be applied. n.d., not determined.

^e^ LPS, lipopolysaccharide.

^f^ PS, polysaccharide.

**FIG 1:**
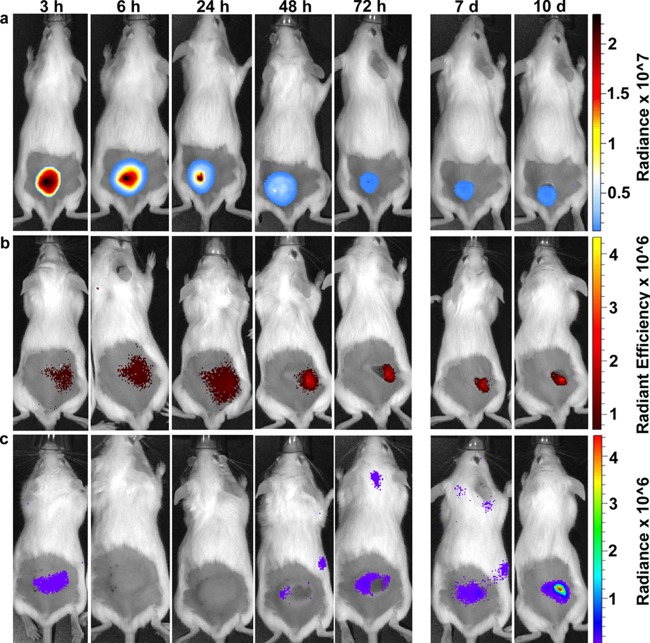
*In vivo* tracking of *P. aeruginosa* LESB58 infection. The infection progress was monitored at the indicated time points (from 3 h to 10 days postinfection). CD-1 mice were injected with a high number of bacteria (5 × 10^7^ CFU). (a) *In vivo* tracking of bioluminescently labeled (live) bacteria showed stable expression of the reporter gene that slowly decreased over time. While a high signal was observed 3 h postinfection, the intensity after 6 h got weaker and was more extensively spread around the tissue swelling. Starting on day 3, the signal was focused at the visible dermonecrotic region. Decreasing luminescence in the first 3 days, despite a modest 3- to 10-fold increase in bacterial numbers, might have been due to decreased metabolic activity under the conditions of the high-density infections, which would influence energy-dependent enzyme activity and/or plasmid copy number. (b) Mice were injected i.v. with a fluorescent neutrophil-specific NIR probe. The signal was scattered around the injection site until day 1 and focused at the necrotic tissue from day 2 onward. (c) ROS and RNS production was tracked using the chemiluminescent probe L-012 (injected between the ears). ROS/RNS production was turned on within 3 h postinfection, rapidly disappeared after 6 h, and reappeared after 24 h. (a to c) Mice were imaged using an *in vivo* image system (IVIS) for a maximum of 10 days. The experiment was repeated at least three times with a minimum of two mice/group.

We therefore tested a cystic fibrosis (CF) isolate since it is known that one of the frequent adaptations to chronic growth in the CF lung is a decrease in motility due to decreased production or an absence of flagella (13). The LESB58 *P. aeruginosa* Liverpool epidemic strain is a well-characterized, highly transmissible isolate that causes chronic CF lung infections that do not disseminate (14, 15). When injected under the thin skeletal muscle at the right dorsum, this strain caused chronic infection in the host for up to 10 days (Fig. 1), with no mortality at infectious doses of up to 10^9^ organisms per mouse. Furthermore, we were unable to recover bacteria from various body organs, indicating that bacteria persisted in high numbers near the injection site and did not disseminate into other body organs. We confirmed here the characteristics of poor swimming motility on semisolid (0.3%) nutrient agar plates (motility zone, ~18%) and relatively slow growth (doubling time in nutrient broth, ~50%) of strain LESB58 compared to strains PA14 and PAO1 (15).

### *In vivo* growth, persistence, and survival of *P. aeruginosa* LESB58 during cutaneous infection.

To demonstrate the ability of LESB58 to initiate and establish chronic abscess infections in a murine model, we tracked bacterial infection and proliferation in real-time *in vivo* using a bioluminescence-tagged strain, LESB58(pUCP.lux) (Fig. 1a), and further determined bacterial counts for up to 10 days postinfection. At an input dose of <10^6^ CFU, strain LESB58 did not establish a long-term infection. However, at an input dose of 5 × 10^7^, this strain survived very well subcutaneously within a raised lump (abscess) and caused an area of local dermonecrosis (70 mm^2^ ± 20 mm^2^) at the skin surface above the abscess. Luminescence decreased from day 1 until it reached a stable level at day 3 and remained at this level until around day 10. However, energy-dependent luminescence can be influenced by bacterial metabolic activity and/or metabolism-sensitive plasmid copy number, which might have been reduced under the conditions of high-density infections. Therefore, we also harvested bacteria directly from the subcutaneous abscess. These data showed that strain LESB58 grew over the first 3 days by 2- to 10-fold, from a starting inoculum of 5 × 10^7^ CFU to a level of 1 to 5 × 10^8^ CFU/mouse. This increase was very consistent and reproducible in all experiments (using a total of more than 30 mice), and the level of infection was subsequently maintained unchanged for a further 7 days. Around day 10, the bacterial load started to decrease by approximately 1- to 5-fold below the original injection inoculum level, and the scab from the dermonecrotic lesion subsequently fell off.

### Real-time tracking of the inflammatory response and histological evaluation.

**FIG 2:**
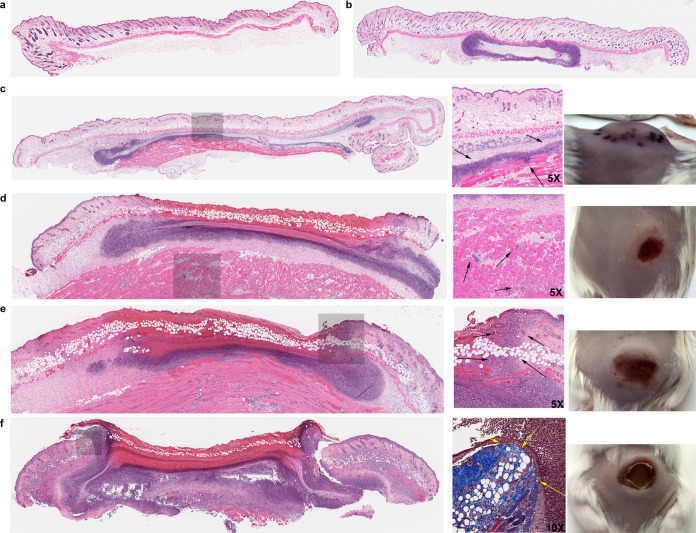
Histological sections and skin images of *P. aeruginosa* cutaneous infection on female CD-1 mice over several days. (a) Uninfected mouse skin tissue (including epidermis, dermis, adipose tissue, and thin skeletal muscle/panniculus carnosus). (b) Injection bubble underneath thin skeletal muscle surrounded by neutrophils and macrophages. (c to f) Infection progress after (c) 1, (d) 2, (e) 3, and (f) 7 days. Images show injected area with a zoom window (gray area), magnified location, and skin appearance (left to right). (c) On day 1, abundant neutrophils and macrophages were observed around the infection site that penetrated into the deep skeletal muscle tissue (arrows show deep inflammation and skeletal muscle damage). (d) Day 2 showed the extension of inflammation to the skeletal muscle (arrows) and necrosis of the overlying dermis. (e) On day 3, the inflammation extended further into the upper dermis (arrows). (f) On day 7, a large abscess with overlying crust/scab was formed. Adjacent reparative changes with granulation tissue, fibroblastic reaction, and epidermal reepithelialization were evident. Magnification shows Masson’s trichrome staining, highlighting fibroblastic proliferation around the edge of the necrotic tissue (yellow arrows).

The host inflammatory response to microbial intruders includes the chemoattraction and activation of neutrophils and macrophages to eradicate invading pathogens in part through the generation of respiratory burst products (including peroxynitrite and superoxide anions) (16). Initial control of local infections generally involves rapid neutrophil infiltration into the infection site, which we observed visually using a neutrophil-specific probe, NIR, a cyanine7-conjugated, polyethylene glycol (PEG)-modified hexapeptide that specifically binds the formylpeptide receptor (FPR) of neutrophils (16). As shown in Fig. 1b, neutrophils were rapidly recruited to the inflammatory site within the first 3 h after injection of bacteria and were present throughout the infection period, becoming more localized and concentrated at the site of infection at day 2. Histological evaluation confirmed the presence of abundant neutrophils and macrophages around the injection site from the early onset of the infection through day 10 (Fig. 2). Thus, despite the recruitment of phagocytic cells to the infected tissue, bacteria persisted inside the abscess.

Since reactive species can be produced by phagocytic cells and can reach very high concentrations during inflammation, we assessed their production using an L-012 chemiluminescence probe (17) to monitor the generation of a respiratory burst over several days. ROS/RNS production was very high within the first 3 h postinfection (indicating the influx of phagocytic cells and an early respiratory burst) but had almost disappeared by 6 h (Fig. 1c), with virtually no detectable luminescence at 24 h. However, ROS/RNS levels slowly increased commencing on the second day (starting from the edges of the inflamed region where tissue necrosis first became visible) and by day 7 had reached levels similar to those observed on day 1, with the highest ROS/RNS levels being detected at day 10 (Fig. 1c). The rapid induction of ROS within the first few hours was likely due to the observed rapid influx and activation of neutrophils, although skin keratinocytes and tissue macrophages could have played a role. The subsequent rapid (within 3 h) decline of ROS levels indicated that activation was transient, enabling the establishment of a chronic infection.

Histological investigations performed by an independent expert indicated that an inflammatory infiltrate penetrated into the deep skeletal muscle tissue within the first 24 h, thereby causing local tissue swelling, increased tension, and induration (Fig. 2b). On day 2, the disease further progressed into the deep skeletal muscle area and, around the necrotic tissue, dermal neutrophil infiltration and a large amount of cell debris were apparent (Fig. 2c). On day 3, inflammation extended further into the upper dermis and the underlying muscle tissue started to push, to the surface, the early abscess/pus as observed by the accumulation of cellular debris. Moreover, starting on days 3 and 4, mild fibroblastic proliferation at the edges of the necrotic tissue could be observed that further developed into extensive fibroblastic proliferation, tissue granulation, and reepithelialization with crust/scab formation on day 7 (Fig. 2d). At that time, on day 7, abundant neutrophils and macrophages were still present within and around the abscess/pus tissue. The visible scab that formed on the surface of the skin contained many bacteria. By 10 days, subsequent reepithelization eventually removed the crust, the number of bacteria was reduced, and the abscess started to resolve; the scab fell off around day 14.

### Therapeutic treatment of cutaneous abscesses.

Skin abscesses are commonly treated by surgical incision of the infected tissue to release the accumulated pus through drainage. Antibiotics are often prescribed because drainage does not result in the complete removal of bacteria in many cases. Here, we treated *Pseudomonas* abscess infections with clinically relevant antibiotics, including the aminoglycoside gentamicin (Gm), the quinolone ciprofloxacin (Cip), and the carbapenem meropenem (Mero). The MIC values determined for the LESB58 strain by the broth microdilution assay in three or more independent experiments were 15.6 μg/ml for gentamicin, 1.6 μg/ml for ciprofloxacin, and 1.6 μg/ml for meropenem.

The antibiotics were initially administered intravenously (i.v.) at a high dose (30 mg/kg of body weight, i.e., 2- to 6-fold the average daily dose for skin and soft tissue infections [SSTIs] [18, 19]) once daily. Using this route of delivery, we observed only a minor reduction in CFU counts with ciprofloxacin and no significant change in average abscess lesion sizes (Fig. S2). These data indicate that this is a stringent model for testing antibiotic treatment of high-density infections.

10.1128/mBio.00140-17.2 FIG S2  Therapeutic treatment (i.v. injection) of *P. aeruginosa* LESB58 infection. CD-1 mice were infected with 5 × 10^7^ *P. aeruginosa* LESB58. Mice were treated with saline solution, gentamicin (30 mg/kg), or ciprofloxacin (30 mg/kg) 1 h post-bacterial infection and subsequently once daily for the next 48 h. Lesion sizes and CFU counts were determined 3 days postinfection. The experiment was repeated at least twice. Dermonecrosis measurements (a) and CFU counts/abscess (b) showed no significant differences. Download FIG S2, TIF file, 0.6 MB. Copyright © 2017 Pletzer et al. 2017 Pletzer et al. This content is distributed under the terms of the Creative Commons Attribution 4.0 International license.

**FIG 3:**
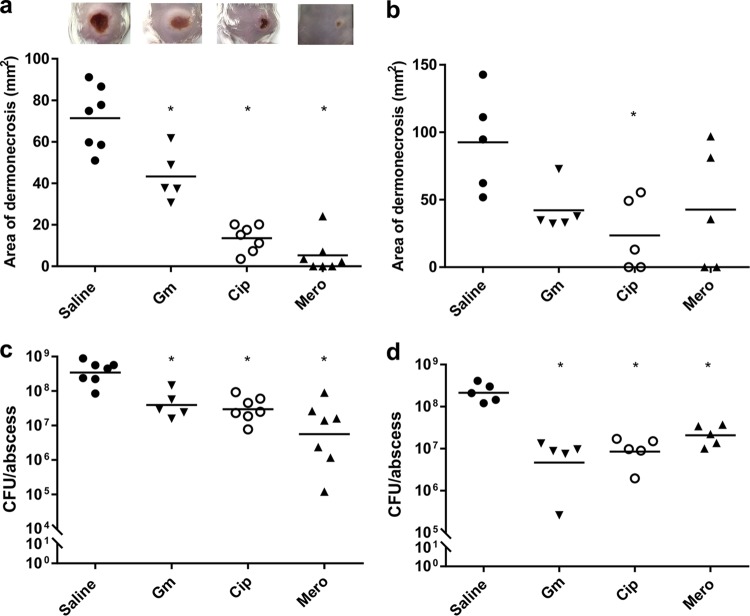
Therapeutic treatment of mouse cutaneous abscesses. CD-1 mice were infected with 5 × 10^7^ CFU *P. aeruginosa* LESB58 and treated with saline solution, gentamicin (Gm; 8 mg/kg; 200 µg/abscess), ciprofloxacin (Cip; 0.4 mg/kg; 10 µg/abscess), or meropenem (Mero; 0.4 mg/kg; 10 µg/abscess). Lesion sizes and CFU counts were determined 3 days postinfection. The experiment was repeated at least twice. (a and b) Dermonecrosis measurements. (c and d) CFU counts/abscess. (a and c) Single antibiotic application 1 h post-bacterial infection. (b and d) Multiple antibiotic applications were performed starting from 1 h post-bacterial infection and subsequently every day for another 48 h. *, significant difference from saline solution-treated mice (one-way ANOVA; *P* < 0.01).

This led us to attempt a more direct therapeutic approach wherein antibiotics were injected straight into the inflamed tissue region. Thus, 1 h after the initiation of bacterial infection, we performed intra-abscess (i.a.) treatment with gentamicin (8 mg/kg; 200 μg/abscess), ciprofloxacin (0.4 mg/kg; 10 μg/abscess), or meropenem (0.4 mg/kg; 10 μg/abscess). This single intra-abscess antibiotic dose was able to significantly reduce or completely inhibit superficial tissue dermonecrosis (Fig. 3a). However, the tested antibiotics were unable to eradicate the bacterial burden and showed a reduction in CFU of only about 10- to 100-fold (Fig. 3c). This observation prompted us to investigate bacterial clearance in the skin tissue after daily antibiotic treatment. Remarkably, daily antibiotic injections into the inflamed area, at the concentrations described above, did not eradicate bacteria. Indeed, multiple injections reduced abscess sizes and reduced the CFU by more than 10-fold (Fig. 3b and d) but did not have significantly greater efficacy than a single treatment dose.

### *P. aeruginosa* caused similar levels of chronic infection in mice that differed by age, gender, and genetic background.

**FIG 4:**
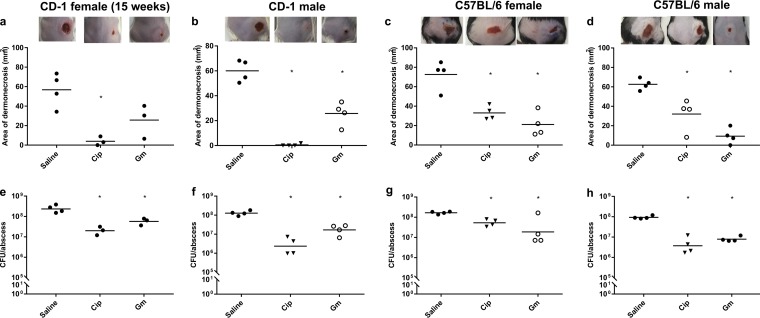
Infection and therapeutic treatment of other mouse strains. All mice were infected with 5 × 10^7^ CFU *P. aeruginosa* LESB58 and treated with saline solution, gentamicin (Gm; 8 mg/kg; 200 µg/abscess), or ciprofloxacin (Cip; 0.4 mg/kg; 10 µg/abscess) 1 h post-bacterial infection. Lesion sizes and CFU counts were determined 3 days postinfection. The experiment was repeated at least twice. (a to d) Dermonecrosis measurements. (e to h) CFU counts/abscess. (a and e) Fifteen-week-old CD-1 female mice. (b and f) Seven-week-old CD-1 male mice. (c and g) Seven-week-old C57BL/6 female mice. (g and h) Seven-week-old C57BL/6 male mice. *, significant difference from saline solution-treated mice (one-way ANOVA, *P* < 0.01).

The experiments described above used 7-week-old female CD-1 mice. Therefore, we considered the effect of other mouse-related factors such as age, gender, and genetics. Many infection models do not work well in older animals, which tend to be more resistant to infection (20). However, the LESB58 strain caused infection in 15-week-old CD-1 mice that was similar to that seen in 7-week-old mice, with abscess sizes approximately 10 mm^2^ smaller than those seen in the younger mice, and that resulted in very similar bacterial loads inside the abscesses after 3 days (Fig. 4a and e).

Gender and sex steroids also play important roles in mouse skin morphology and physiology (21), as well as in infection dynamics and immune responses (22). Thus, we tested whether gender had an impact on our infection model. Seven-week-old CD-1 male mice weighed about the same as 15-week-old female mice. Correspondingly, 7-week-old male CD-1 mice formed abscess lesions that were similar in size to those of 15-week-old CD-1 female mice and only slightly smaller than those of 7-week-old CD-1 female mice. In addition, the antibiotic treatments showed similar results (Fig. 4b and f).

It has been suggested that the immune response in inbred mice is significantly different from that of outbred mice (23) (affecting, for example, CD8^+^ T cell responses to bacterial infection [24]). We thus investigated whether our infection model could be applied to inbred C57BL/6 mice. Seven-week-old C57BL/6 mice weighed on average 25% less than outbred CD-1 mice. However, neither genetics nor weight nor size differences influenced abscess formation (Fig. 4c to h). In addition, similar bacterial inocula could be applied to both mouse strains (regardless of their gender) and antibiotics worked in similar fashions. As with the CD-1 mice, male C57BL/6 mice had abscesses that were smaller (by approximately 10 mm^2^) than those of their female counterparts.

### Other Gram-negative pathogens caused subcutaneous infections.

**FIG 5:**
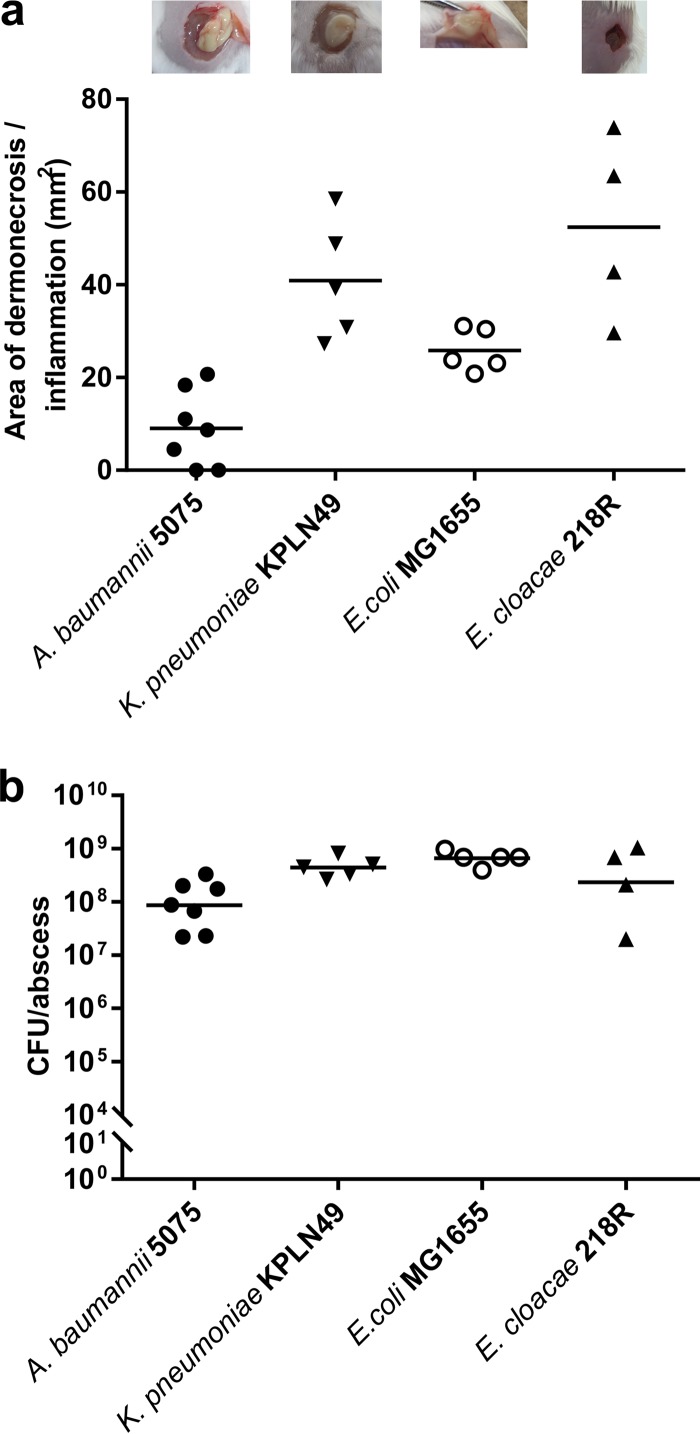
Mouse cutaneous infection with Gram-negative bacteria. CD-1 mice were infected with 5 × 10^8^ CFU *A. baumannii* AB5075, *K. pneumoniae* KPLN49, or *E. coli* K-12 MG1655 as well as with 5 × 10^7^ CFU *E. cloacae* 218R1. Abscess sizes and CFU counts were determined 3 days postinfection. The experiment was repeated at least twice. (a) Abscess formation and inflammation (i.e., accumulated infiltrate under the skin) showed large pus cavities for *A. baumannii*, *K. pneumoniae*, and *E. coli*, while *E. cloacae* formed a dry abscess (top panels). Abscess measurements are shown below the top panels. (b) CFU counts/abscess.

Gram-negative pathogens often cause long-term or chronic infections, and such infections are increasingly difficult to treat due to multidrug and adaptive resistance (1, 25). Therefore, we tested whether our model could be extended to other bacteria. It was shown that multidrug-resistant *A. baumannii* strain Ab5075, wild-type *K. pneumoniae* strain KPLN49, β-lactamase-overproducing *E. cloacae* strain 218R1, and commonly used laboratory strain *E. coli* K-12 MG1655 were able to establish long-term cutaneous infections in CD-1 mice (Fig. 5). *A. baumannii* AB5075, *K. pneumoniae* KPLN49, and *E. coli* MG1655 were injected at 5 × 10^8^ CFU per mouse. Increasing the inoculum did not result in larger abscesses or have any impact on mortality, while lowering the inoculum to <10^8^ CFU increased the inconsistency of the data as well as clearance of the bacteria. *E. cloacae* 218R1 was injected at 5 × 10^7^ CFU, which resulted in consistent and reproducible data. However, increasing the inoculum to >10^8^ CFU resulted in increased abscess sizes and led to mice reaching their humane endpoint within 48 h. Lower inocula (<10^7^ CFU) yielded smaller abscess sizes and increased the inconsistency of the data. Only *E. cloacae* formed a dry abscess, but the 3 other strains caused a raised lump due to the presence of bacteria and pus under the skin. The infection persisted for 3 days, with numbers of viable bacteria similar to those of the bacteria used for the injection inoculation (1 × 10^8^ to 5 × 10^8^ organisms), indicating that our model could be used to study other important Gram-negative pathogens.

As with *P. aeruginosa*, these effects were at least somewhat strain specific and pilot experiments showed that wild-type strain *A. baumannii* ATCC 17987, drug-resistant isolate *A. baumannii* SENTRY C8 (26), wild-type *K. pneumoniae* ATCC 13883, and multidrug- and polymyxin-resistant isolate *K. pneumoniae* KPC1825971 (27) were unable to cause an infection even at high (10^9^) inocula. On the other hand, the wild-type *E. coli* O157:H7 (28) strain caused a high mortality rate similar to that seen with strain PA14 when inocula greater than 10^9^ CFU were used. Inocula lower than 10^9^ resulted in strong inconsistencies in abscess formation, mortality, and recovered CFU.

### Virulence of *P. aeruginosa* in the abscess model.

To test whether this model could also be utilized for studying virulence-associated phenotypes, we first investigated the expression of various virulence-associated genes in *P. aeruginosa* LESB58 isolated from the abscess 3 days postinfection. Bacterial RNA was isolated directly from the infected tissues, and we used quantitative real-time PCR (qRT-PCR) to compare the levels of expression of selected genes in abscesses to the levels of expression of the same genes in cultures grown under *in vitro* rich broth conditions (Table 2). Certain virulence factor genes were highly (>5-fold) upregulated, including those involved in pyochelin- and pyoverdine-mediated iron uptake, pilus-mediated adherence, exotoxin A and lipase production, lipopolysaccharide O-antigen chain length, type III secretion regulation, alginate and Psl biofilm matrix production, and motility/chemotaxis. In contrast, genes encoding a global transcriptional response regulator (GacA), the quorum sensing regulators RhlR and LasR, phenazine biosynthesis gene product PhzA2, and the biosurfactant rhamnolipid pathway component RhlB were downregulated (Table 2).

**TABLE 2:** Relative fold changes of *P. aeruginosa* LESB58 mRNA transcripts from abscesses 3 days postinfection^a^

Gene	Description	Function or product	Fold change^b^
*pvdE* (PA2397)	Pyoverdine siderophore ABC transporter	Iron uptake	124.8
*toxA* (PA1148)	Exotoxin A type II secretion system substrate	Exotoxin A	24.9
*wzz* (PA3160)	Lipopolysaccharide (LPS) O-antigen chain length regulator	Surface LPS	24.4
*fimT* (PA4549)	Type 4 pilus biogenesis protein	Adherence	17.9
*lipC* (PA4813)	Lipase type II secretion system substrate	Lipase C	13.2
*lipA* (PA2862)	Major lipase type II secretion system substrate	Lipase A	10.4
*chpE* (PA0417)	Probable chemotaxis protein	Chemotaxis	8.2
*pchB* (PA4230)	Pyochelin siderophore biosynthesis	Iron uptake	8.2
*exsA* (PA1713)	Type III secretion transcriptional activator	Type III secretion	8.0
*mucC* (PA0765)	Positive regulator for alginate biosynthesis	Exopolysaccharide	6.8
*fliI* (PA1104)	Flagellum-specific ATP synthase	Motility	5.4
*pslD* (PA2234)	Psl exopolysaccharide biosynthesis	Biofilm matrix	5.4
*relA* (PA0934)	Stringent stress response mediator ppGpp synthesis	Stringent response	1.2
*nirQ* (PA0520)	Denitrification regulatory protein	N metabolism	−1.2
*algR* (PA5261)	Global two-component response regulator	Regulator	−1.6
*lasR* (PA1430)	Transcriptional regulator for LasRI system	Quorum sensing	−2.0
*pscI* (PA1722)	Type III secretion export protein	Type III secretion	−2.5
*rhlB* (PA3478)	Rhamnolipid rhamnosyltransferase chain B	Biosurfactant	−2.6
*lasB* (PA3724)	Elastase type II secretion system substrate	Elastase	−2.9
*rhlR* (PA3477)	Transcriptional regulator for RhlRI system	Quorum sensing	−6.9
*phzA2* (PA1899)	Phenazine biosynthesis	Phenazine	−12.8
*gacA* (PA2586)	Two-component system response regulator	Global activator	−14.6

^a^ Transcript abundance was determined by quantitative RT-PCR. Data represent the means of results from at least 3 replicates.

^b^ Data represent fold changes compared to cells grown in LB medium.

We also tested the influence of mutations in virulence-associated mutants during infection. Due to the availability of the *P. aeruginosa* PA14 mutant library (29), we used this more virulent PA14 strain as the control and its derived mutants for these experiments. As mentioned above, at a dose of 5 × 10^6^ CFU, PA14 caused a disseminating infection and only 60% of the mice survived after 3 days (Table 1). The importance of iron availability was revealed by the fact that a mutant unable to produce the iron siderophores pyoverdine and pyochelin was unable to cause dermonecrosis, and very few bacteria were found at the injection site after 3 days. The toxin(s) driving dermonecrosis was likely that produced by type III secretion, since dermonecrosis did not occur when mice were infected with a strain with a mutation in *exsA*, encoding a major type III secretion regulator. Intriguingly, the loss of this regulator also caused a 1,000-fold-lower bacterial burden in the area adjacent to the injection site and prevented death and dissemination. In contrast, a strain with a mutation in one of the type III secreted toxins, ExoU, showed more-modest effects on infection in that there was no lethality after 3 days of infection despite its dissemination to the liver and kidneys. A PA14 strain with a mutation in the gene encoding the global regulator AlgR, like the flagellin FliI mutant, caused cutaneous abscesses and dermonecrosis similar to those seen with strain LESB58 but no lethality. It also led to minimal dissemination to the kidney, but not to the liver, and did so in only 1 of 4 mice.

In addition to the results described above, infections with strains with mutations in genes involved in the acyl-homoserine-lactone-dependent quorum sensing systems (*rhlR* and *lasI*) did not lead to mortality in mice but only the strains with a *rhlR* mutation caused a lower bacterial burden in the abscess. Conversely, a strain with a mutation in the *bfiS* gene, encoding a sensor kinase that controls biofilm formation and dispersal, led to strongly increased virulence as reflected by larger abscesses, higher bacterial counts, and strong dissemination and lethality.

## DISCUSSION

Current murine models of chronic infections tend to be technically very challenging to perform and difficult to track without sacrificing animals (9). Here, we have overcome both obstacles by applying a modestly invasive procedure (i.e., subcutaneous bacterial injection into the dorsum on the back of mice) using *P. aeruginosa* cystic fibrosis epidemic isolate LESB58 as an etiologic organism to mimic a chronic abscess infection. At least one reason for the suitability of this strain appeared to be its poor swimming motility, which is consistent with the observation (Table 1) that a *fliI* mutant of strain PA14 caused similar nonlethal, nondisseminating cutaneous abscesses and dermonecrotic lesions (cf. the parent PA14 isolate) that caused progressive mortality and frequent dissemination to the kidneys and/or liver. It is worth noting that many other chronic infection isolates from the CF lung have defective motility due to decreased production or an absence of flagella (13).

Establishing chronic abscess infections by strain LESB58 involved no special preparation of the bacterial inoculum (such as, e.g., encasing the bacteria in agar or agarose beads or pregrowth of the bacteria as biofilms on devices) and employed simple animal handling procedures with no manipulation (e.g., thermal injury or cyclophosphamide) or surgery of mice. The method enabled a range of bacterial inocula to be used, afforded excellent reproducibility, and was neither self-limiting nor rapidly lethal. Even using more-virulent isolates, we still observed persistent infections in more than one-half of the mice, enabling virulence studies. Furthermore, we observed no major effects due to gender, age, or mouse strain. Thus, this model offers an excellent new alternative to the previous animal models, which are generally more complex or have significant limitations. The model was also successfully extended to a variety of nosocomial Gram-negative bacteria, especially focusing on common isolates, including 3 other ESKAPE pathogens.

Reactive oxygen species and reactive nitrogen species (ROS/RNS) contribute to inflammatory disease progression (30), and prolonged ROS/RNS production through accumulation of macrophages and/or neutrophils at the site of the infection is a characteristic feature of such chronic infections (31). In our model, the course of infection could be easily tracked using imaging methods (Fig. 1). Our study revealed that elevated ROS/RNS levels accompanied skin inflammation, albeit with unusual kinetics involving an initial transient burst followed by a later increase that occurred between days 2 and 10. The retention of high levels of bacteria in the infected tissue occurred despite this, and despite an influx of neutrophils, indicating that the presented model can be used as a chronic inflammation model. Thus, this model has significant advantages over many other models by providing an easy, reliable method for tracking infection without the need to sacrifice animals.

It also enables the modeling of bacterial skin and soft tissue infections (SSTIs) (i.e., infections of the skin, subcutaneous tissue, or even muscle tissue), which are a very common problem (32), contributing to 6.8 million emergency department visits annually in the United States (33). *S. aureus*, including methicillin-resistant *S. aureus* (MRSA), is the most common cause of skin infections, but Gram-negative bacteria are becoming increasingly important as causes of up to 20% of SSTIs worldwide (34), emphasizing the necessity for using broad-spectrum anti-infective strategies (32). SSTIs can lead to cutaneous abscesses that are characterized by an accumulation within the dermis of fluid/pus containing both live and dead bacteria and host cells. Abscesses often lead to severe inflammation and induration before further resulting in open sores (32). Current treatments include surgical drainage and the administration of antibiotics to prevent dissemination, although antibiotic efficacy is limited and recurrence can occur (35). Our model clearly mimics many of these features, and we provide evidence that dermonecrosis at the skin surface is due to the presence of type III-secreted toxins, in the case of *P. aeruginosa* strain PA14, while providing insights into other bacterial determinants of infection (Table 1). Of great interest is our data indicating that excessive virulence also appears to be regulated, since a mutation in gene encoding the two-component sensor kinase BfiS that regulates biofilm development caused complete mortality.

Bacteria in high-density infections are likely highly stressed. Our data indicate that at least one major stressor is iron deficiency since transcriptional data showed that genes involved in pyochelin and pyoverdine siderophore-mediated iron uptake were highly upregulated in strain LESB58 during abscess infections, while a siderophore-deficient PA14 mutant was unable to form chronic or invasive infections. Certain virulence factor genes were also highly (>5-fold) upregulated in LESB58, including those involved in pilus mediated adherence, exotoxin A and lipase production, lipopolysaccharide O-antigen chain length, type III secretion regulation, alginate and Psl biofilm matrix production, and motility/chemotaxis. Many of these genes are known to be regulated under iron limiting conditions, suggesting that iron availability strongly influenced infection in this model.

The treatment of high-density chronic infections (e.g., in chronic wounds [36, 37], chronic otitis media [38], lung infections in CF patients [39], etc.) remains very challenging. Adaptive multidrug resistance triggered by *in vivo* growth conditions in densely packed organisms (40) likely limits therapeutic success. It is tempting to speculate that the observed poor efficacy of antibiotics in our abscess model reflects either the high density of bacteria in the abscess or the fact that they were in a highly stressed state. Indeed, there has been some recent evidence suggesting that Gram-positive bacteria isolated from deep tissue abscesses are embedded in biofilm-like matrices (41). Consistent with this, *pslD*, a gene involved in exopolysaccharide biosynthesis, and *mucC*, a positive regulator gene for the synthesis of the exopolysaccharide alginate, were upregulated 5- to 7-fold. Given the high level of adaptive multidrug resistance that accompanies both biofilm formation and stationary-phase bacterial growth, and the common modes of infections exhibiting such adaptations in the clinic, we submit that our model has the potential to enable secondary (i.e., confirmatory animal model) screening for a new generation of antibiotics that are able to overcome such recalcitrant high-density infections.

The development of new antimicrobial agents to fight emerging multidrug resistant bacteria is a crucial step in combating public health challenges. However, while acquired resistance is of great importance, it does not account for all therapeutic failures. The lack of clinically available agents that were designed to address chronic biofilm and abscess infections is important in this regard (25), although cationic anti-biofilm peptides show some promise (42). The validation of animal models that can be used for screening and that reflect more-recalcitrant infections is a pivotal step in bridging the gap to clinical trials for new agents that overcome adaptive resistance. Before novel drug compounds can be even considered for clinical trials, they must undergo detailed *in vitro* and *in vivo* testing to provide adequate information about their mode of delivery, formulation, and efficacy. Here, we have demonstrated an animal model that can be used as a rapid and easy *in vivo* secondary screening assay for novel compounds, enabling toxicity studies and efficacy against a variety of Gram-negative bacteria.

## MATERIALS AND METHODS

### Bacterial strains and growth conditions.

The bacterial strains used in this study are listed in Table S1 in the supplemental material. All organisms were cultured at 37°C in LB or double yeast tryptone (dYT) medium with shaking at 250 rpm. Cultures harboring individual vectors were supplemented with 15 µg/ml gentamicin for *E. coli*, 50 µg/ml gentamicin for *P. aeruginosa* strain PA14, and 500 µg/ml gentamicin for *P. aeruginosa* strain LESB58. Bacterial growth was monitored using a spectrophotometer at an optical density of 600 nm (OD_600_).

10.1128/mBio.00140-17.3 TABLE S1  Bacterial strains used in this study. Download TABLE S1, DOCX file, 0.03 MB. Copyright © 2017 Pletzer et al. 2017 Pletzer et al. This content is distributed under the terms of the Creative Commons Attribution 4.0 International license.

### Construction of the PA14 pyochelin/pyoverdine double mutant.

The construction of the knockout vectors was based on the protocol by Zumaquero et al. (43) and carried out as previously described (44). Briefly, primers pchA-A1/pch-A2 and pchD-B1/pchD-B2 were used to amplify the knockout alleles for the pyochelin genes and primers pvdG-A1/pvdG-A2 and pvdL-B1/pvdL-B2 for the pyoverdine genes (see Table S2). The obtained A and B fragments were used in an overlapping PCR with A1 and B2 primers. Next, each fusion fragment was cloned into suicide vector pEX18Gm (45) via the use of EcoRI/HindIII restriction sites and verified by sequencing.

10.1128/mBio.00140-17.4 TABLE S2  Primers used in this study. Download TABLE S2, DOCX file, 0.01 MB. Copyright © 2017 Pletzer et al. 2017 Pletzer et al. This content is distributed under the terms of the Creative Commons Attribution 4.0 International license.

The generation of the PA14 siderophore mutant was done stepwise, deleting first the pyochelin cluster (3.3 kb) and subsequently the pyoverdine cluster (13.0 kb). The method was based on the site-specific insertional mutagenesis strategy of Schweizer and Hoang (46) and carried out as described previously (44). In order to confirm the deletions, locus-specific primers that bind up- and downstream of the operons were used (pchA_out1/pchD_out2 and pvdG_out1/pvdL_out2, respectively) and the resulting knockout fragments were verified by sequencing.

### Construction of *Pseudomonas* bioluminescent strains.

The *P. aeruginosa* PA14.lux strain was constructed using a mini-Tn*7* insertion system (47, 48). Briefly, plasmid pUC19 (NEB) was digested with PvuII and the resulting DNA fragment containing the *lac* promoter, a constitutively expressed promoter in *P. aeruginosa*, was cloned into pTOPO Zero-Blunt cloning vector (Invitrogen). This vector was then further digested with Nsil, which enabled the transfer of the *lac* promoter fragment into a PstI-digested pUC18T-mini-Tn-7T-Gm-lux (48) plasmid carrying the bacterial luciferase (*lux*) operon. The resulting plasmid, pUC18T-mini-Tn-7T-Gm-lux.P_lac_, was coelectroporated with helper plasmid pTNS2 (47) (500 ng each) into *P. aeruginosa* PA14 cells as described earlier (49). Positive clones, showing strong bioluminescence, were selected on LB agar plates containing gentamicin and further verified for correct chromosomal insertion by PCR as described previously (47, 48).

Since this single-copy chromosomal insertion of the *lux* operon driven by a constitutive promoter in clinical isolate strain *P. aeruginosa* LESB58 resulted in a weak bioluminescent signal, we constructed a plasmid harboring the *lux* operon under the control of a constitutive promoter (P_lac_). Therefore, we transformed pUCP.lux (50) into LESB58 by electroporation (49), which further enabled constitutive bioluminescence production in live cells. Plasmid stability was verified by restreaking single colonies on agar plates without antibiotic selection for 4 days, which did not lead to the loss of luminescence signals.

### Antibiotics.

Antibiotics (gentamicin [Gm], ciprofloxacin [Cip], and meropenem [Mero]) were purchased from Sigma-Aldrich (United States Pharmacopeia [USP]) (Reference Standard grade). All compounds were dissolved in endotoxin-free water (E-Toxate; Sigma-Aldrich). For *in vivo* experiments, compounds were diluted into 0.9% NaCl (Sigma-Aldrich).

### Drug susceptibility tests.

The MICs of drugs for *P. aeruginosa* LESB58 were determined by the broth microdilution assay in 96-well plates using Mueller-Hinton broth (MHB) (51). All tests were performed in at least triplicate following the Clinical and Laboratory Standards Institute recommendations. Bacterial growth (37°C) was examined by visual inspection after 24 h of incubation. The MIC was defined as the lowest concentration of a compound that completely prevented visible cell growth.

### Study approval and animals.

Animal experiments were performed in accordance with The Canadian Council on Animal Care (CCAC) guidelines and were approved by the University of British Columbia Animal Care Committee (protocol A14-0363). Mice used in this study were either outbred CD-1 (male and female) or inbred C57BL/6 (male and female) mice. All animals were purchased from Charles River Laboratories, Inc. (Wilmington, MA) and were 7 weeks of age at the time of the experiments, except for the experiment in which we tested female CD-1 mice that were 15 weeks of age. Female CD-1 mice weighed about 25 g ± 5 g. Seven-week-old male CD-1 mice and 15-week-old female mice weighed about 35 g ± 5 g. Male and female C57BL/6 mice weighted about 17 g ± 2 g.

### Abrasion model.

*P. aeruginosa* PA14.lux was grown to an OD_600_ of approximately 0.5 in LB broth. Prior to injection, bacterial cells were washed twice with saline solution and resuspended to a final concentration of an OD_600_ of 0.5. In order to establish the abrasion model, we used immunocompetent and immunocompromised CD-1 female mice and bacterial inoculation concentrations of 1 × 10^10^ CFU/ml in the immunocompetent mouse abrasion model and 1 × 10^5^ CFU/ml in the immunocompromised abrasion model. Additionally, for the immunocompromised mouse abrasion model, mice were injected intraperitoneally (i.p.) with 200 mg/kg of body weight of cyclophosphamide 4 and 2 days prior to bacterial infection. On day 0, all mice were anesthetized with i.p. injections of ketamine (65 mg/kg) and xylazine (5 mg/kg) and with 1 to 3% isoflurane to maintain the mice at a surgical plane of anesthesia. The fur was removed from the back of each mouse using clippers, and then a small abrasion of approximately 1 cm^2^ was made using a nail file and tape stripping with bandages until the abrasion was red and glistening. Ten microliters of PA14.lux (corresponding to 1 × 10^7^ or 1 × 10^8^ CFU for immunocompetent mice and 1 × 10^3^ or 1 × 10^5^ CFU for immunocompromised mice) or saline solution was dropped onto the abrasion, and the surface was subsequently allowed to dry fully before the mice recovered from the anesthesia. Mice were observed daily and experiments performed at least twice independently with 2 to 5 animals per group. Mice were euthanized with 120 mg/kg i.p. sodium pentobarbital.

### Cutaneous infection (abscess) model.

The fur on the back of each of the mice was removed by shaving and application of chemical depilatories. All microorganisms used in this infection model were grown to an OD_600_ of 1.0 in dYT broth. Prior to injection, bacterial cells were washed twice with sterile phosphate-buffered saline (PBS) and resuspended to a final concentration, depending on the strain used, as outlined in Table S1. Briefly, for *P. aeruginosa* LESB58 injections, an inoculum of 5 × 10^7^ CFU was used that produced reproducible abscess sizes and bacterial counts. For *P. aeruginosa* PA14 injections, an inoculum of 5 × 10^6^ CFU was used that was responsible for about 40% mortality in this model. *A. baumannii* AB5075, *K. pneumoniae* KPLN49, and *E. coli* MG1655 were injected at 5 × 10^8^ CFU, while *E. cloacae* 218R1 was injected at 5 × 10^7^ CFU.

Bacteria were injected (50 μl) into the right side of the dorsum underneath the thin skeletal muscle. All utilized antibiotics were tested for skin toxicity prior efficacy testing. Antibiotics or saline solution (50 μl) was directly injected subcutaneously into the infected area (intra-abscess [i.a.] injection) at 1 h postinfection and daily when indicated. In addition, we applied antibiotics intravenously 1 h postinfection and subsequently daily throughout the experiment when indicated. The progression of the disease/infection was monitored daily. Abscess lesion sizes (visible dermonecrosis) were measured using a caliper on day 3. Swelling/inflammation was not considered in the measurements. Skin abscesses (including all accumulated pus) were excised and homogenized in sterile PBS using a Mini-Beadbeater-96 cell disrupter (BioSpec Products) for 5 min and bacterial counts (in CFU) determined by serial dilution.

Other body organs such as the kidney (left side), liver (right lobe), spleen, and lymph nodes (mesenteric and inguinal) were harvested when indicated to determine whether the infection had disseminated. Experiments were performed at least twice independently with 2 to 5 animals per group. Mice were euthanized with carbon dioxide.

### Tracking luminescence-tagged bacteria during infection.

To follow disease progress in real time, we used bioluminescently labeled *P. aeruginosa* strains. Bioluminescence images were acquired (60 s exposure, medium binning) at different times after the initiation of infection by using a Lumina *in vivo* image system (IVIS) (PerkinElmer, Waltham, MA) and analyzed using Living Image software.

### Reactive oxygen species and reactive nitrogen species (ROS/RNS).

In order to detect the production of ROS/RNS generated by phagocytes during the inflammation, we used a chemiluminescence probe, L-012, that has high sensitivity to superoxide and peroxynitrite anions (17). The probe (25 mg/kg) was subcutaneously injected between the ears of the mice at various time points during the course of the infection. The highest luminescence signals were obtained within 20 ± 2 min after injecting the probe. Representative images were acquired using a Lumina IVIS (60 s exposure, medium binning) and analyzed using Living Image software.

### *In vivo* neutrophil tracking.

Neutrophil chemotaxis and activation were measured and visualized using NIR (Kerafast), a neutrophil-specific fluorescence imaging agent that specifically binds to the formylpeptide receptor of neutrophils. The probe was applied via intravenous injection at 100 nmol/kg, and activated neutrophils which had been recruited to the inflammation site were imaged (60 s exposure, medium binning) using a Lumina IVIS (excitation, 745 nm; emission, 800 nm) and analyzed using Living Image software. Images were subjected to adaptive background subtraction, and the fluorescence emission was normalized to the incident excitation intensity (radiance of the subject/illumination intensity).

### Histology.

For histopathology studies, the inflamed region or abscess (including the skin surrounding the abscess) was excised and fixed in 10% buffered formalin. Cross-sectioning (3 sections from different areas of the abscess) and histochemical staining (hematoxylin and eosin [H&E], Masson’s trichrome, and Gram staining) were performed by Wax-it Histology Services Inc. (University of British Columbia, Vancouver, Canada). Image slide analysis was independently performed in a single-blind manner by pathologist Hamid Masoudi (Vancouver Coastal Health). Histological evaluation was performed daily, and representative images are shown.

### RNA isolation.

*In vitro* cultures were grown overnight in dYT broth, diluted to an OD_600_ of 0.1, and further grown until the mid-logarithmic phase (OD_600_ of approximately 0.5). Samples were resuspended in bacterial Protect reagent (Qiagen) and harvested by centrifugation, the supernatant was decanted, and the pellet was frozen at −80°C. Total RNA was isolated using an RNeasy minikit (Qiagen) following the manufacturer’s instructions. The obtained RNA was treated with DNase (Ambion/Life Technologies) and subsequently quantified using a Nanodrop 2000 spectrophotometer (Thermo Fischer Scientific) and RNA quality determined using a model 2100 Bioanalyzer (Agilent).

*In vivo* skin abscess tissues (up to 100 mg/mouse) were excised as described above, immediately stored in RNAlater (Qiagen) supplemented with 1 μl/ml SUPERase•In RNase inhibitor (Thermo Fisher Scientific), and subsequently frozen at −80°C. Prior to maceration of the tissue samples in liquid nitrogen, samples were incubated for 10 min at 23°C in storage RNAlater by adding a final concentration of 0.05% Triton X, 10 U/ml RNA inhibitor (RNAsin; Promega) (N211A), 1 mM dithiothreitol (DTT), 200 μg DNase I, and 500 μM MgCl_2_). After the samples were mashed, frozen tissue powder was transferred into TRIzole (Ambion) (1 ml/100 mg). The mixture was loaded onto QIA shredder columns (Qiagen) and centrifuged for 10 min (13,000 rpm/4°C). Chloroform was subsequently added to the flowthrough, and the mixture was shaken vigorously and incubated for 3 min. Then, samples were centrifuged at 13,000 rpm and 4°C for 15 min and the aqueous phase of the sample was carefully transferred into new tubes. RNA was precipitated using 100% isopropanol and 75% EtOH. The RNA obtained was DNase I treated as described above, and RNA samples were applied to a MicrobEnrich kit (Ambion) to remove the large quantity of contaminating mammalian RNA and further analyzed using a model 2100 Bioanalyzer (Agilent).

### Quantitative real-time (qRT) PCR.

High-quality RNA was reverse transcribed and amplified with a Roche LightCycler 96 instrument in combination with a Qiagen OneStep RT-PCR kit according to the protocols of the manufacturers. Template RNA (5 ng/sample) was used in a standard 25-μl qRT-PCR reaction mixture with specific primers (Table S2). Each sample was analyzed for gene expression in at least triplicate. Quantification of mRNA transcripts was performed by the comparative threshold cycle (*C*_*T*_) method (52). The 16S gene was used as a normalizer.

### Testing mutants of *P. aeruginosa.*

Various *P. aeruginosa* PA14 mutants deficient in virulence genes (according to the virulence factor database [mgc.ac.cn]) were chosen from the PA14 transposon insertion library, cultured in dYT broth, and adjusted to a CFU count of approximately 5 × 10^6^ CFU/injection (Table S1). The infection was done as described above, and animals were monitored for mortality/health twice a day for up to 3 days. On day 3, the mortality, average dermonecrosis, abscess bacterial burden (as CFU), and numbers of animals with bacteria disseminating into the kidney and liver of surviving animals were assessed.

### Statistical analysis.

Statistical evaluations were performed using GraphPad Prism 6.0 (GraphPad Software, Inc., La Jolla, CA). *P* values were calculated using one-way analysis of variance (ANOVA), a two-tailed unpaired Student’s *t* test, a two-tailed Fisher’s exact test, or a two-tailed Mann-Whitney test (data were considered significant when *P* values were below 0.01 or 0.05 as indicated).
