# Supplementary figures and images for: New Mouse Model for Chronic Infections by Gram-Negative Bacteria Enabling the Study of Anti-Infective Efficacy and Host-Microbe Interactions
Source: mBio. 2017 Feb 28;8(1):e00140-17. doi: 10.1128/mBio.00140-17 (PMC5347345; doi:10.1128/mBio.00140-17)

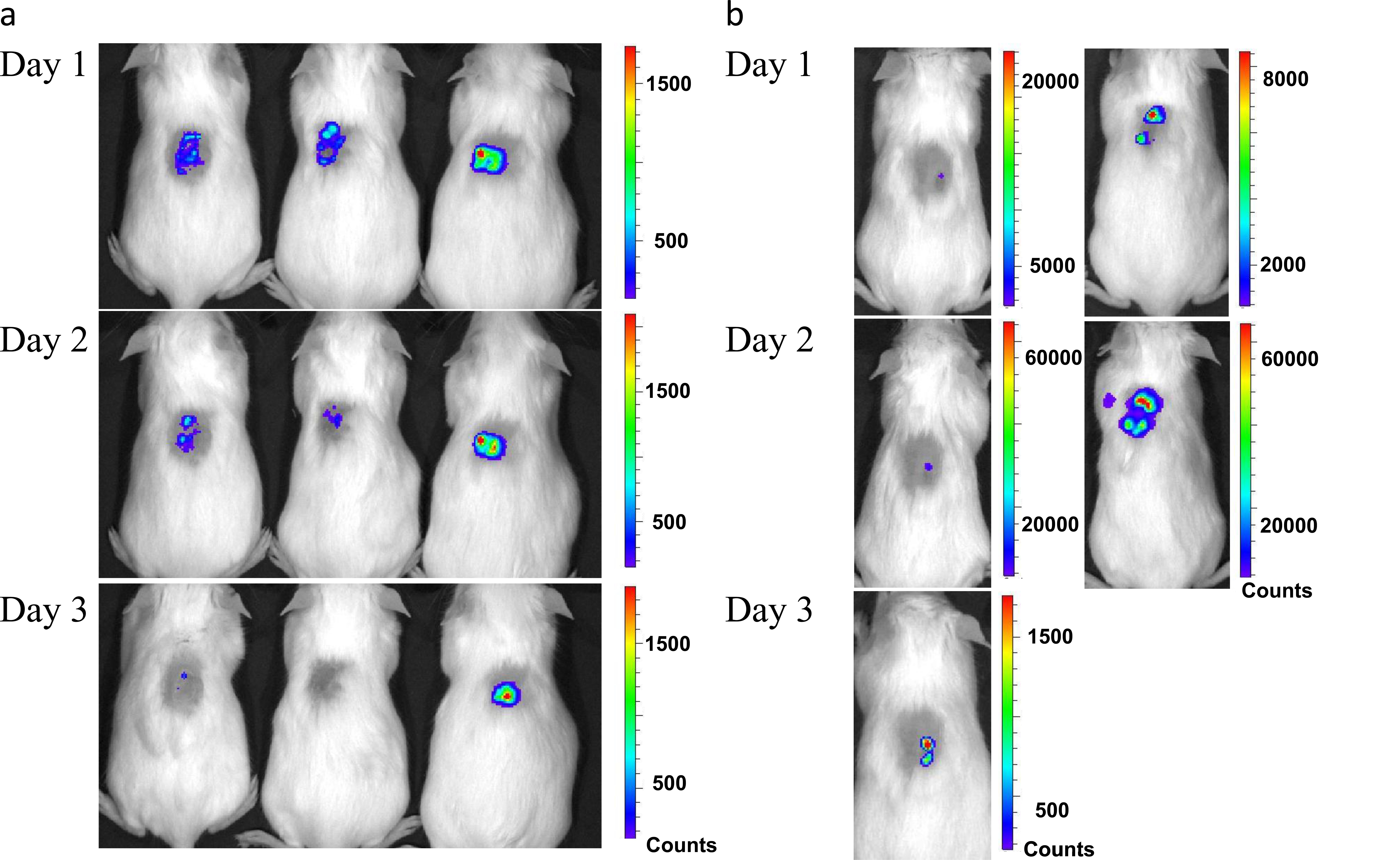

Supplement: FIG S1 [file mbo001173222sf1.tif]

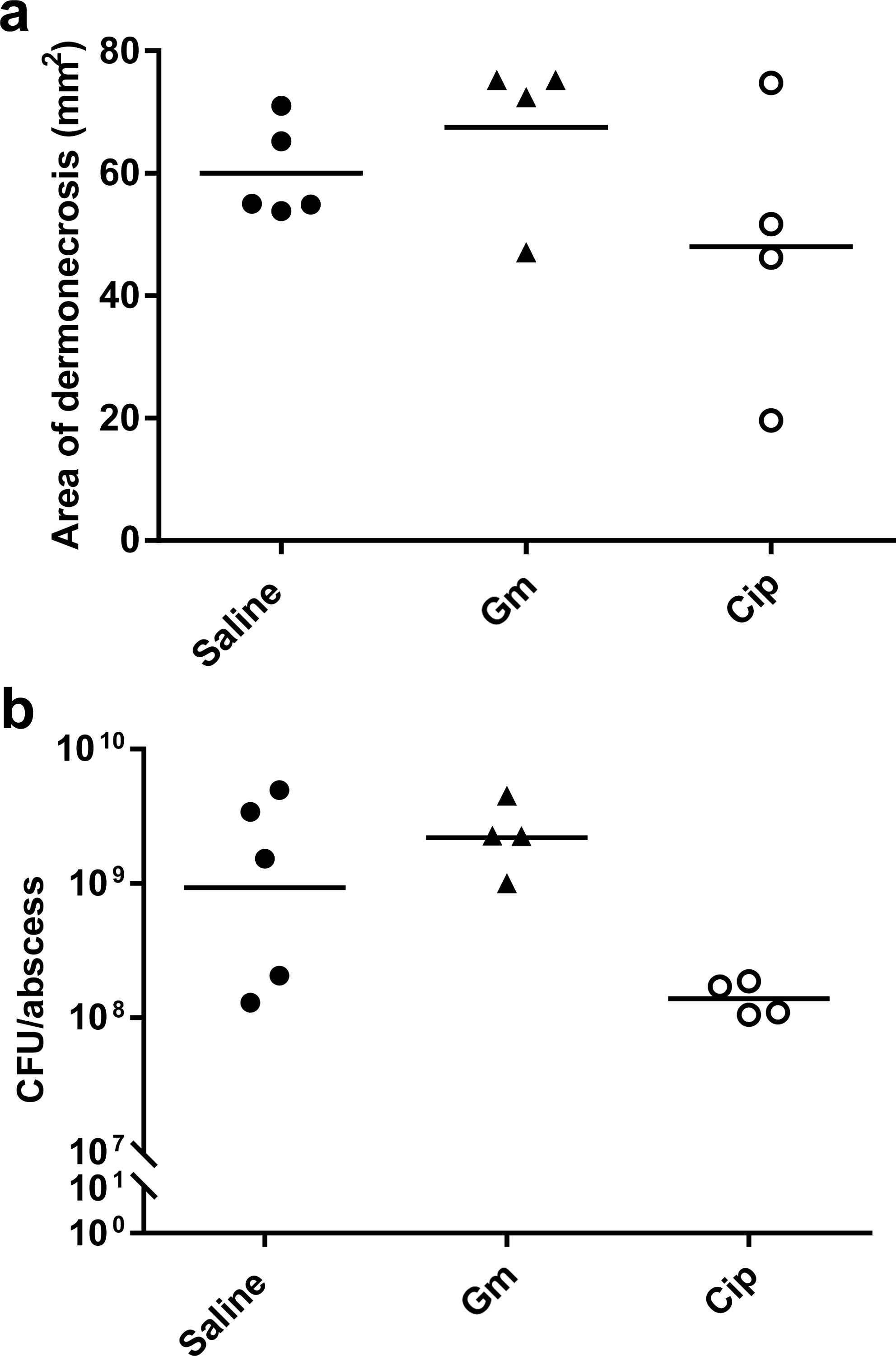

Supplement: FIG S2 [file mbo001173222sf2.tif]
