# Supplementary material for: New Mouse Model for Chronic Infections by Gram-Negative Bacteria Enabling the Study of Anti-Infective Efficacy and Host-Microbe Interactions
Source: mBio. 2017 Feb 28;8(1):e00140-17. doi: 10.1128/mBio.00140-17 (PMC5347345; doi:10.1128/mBio.00140-17)
Supplement: TABLE S1 [file mbo001173222st1.docx]

# Table S1: Bacterial strains used in this study

| **Strain** | **Relevant characteristics or genotype^a^** | **Injection inoculum^b^** | **Reference or source^c^** |
| --- | --- | --- | --- |
| ***Pseudomonas aeruginosa*** |  |  |  |
| LESB58 | Liverpool Epidemic Strain isolate | 5 × 10^7^ | (1) |
| PA14 | Laboratory wild type strain | 5 × 10^6^ | (2) |
| PA14.Δ*pchAD/pvdGL* | *pchAD/pvdGL* operon deletion mutant | 5 × 10^6^ | This study |
| PA14.*algR*::MAR2xT7 | *algR* transposon mutant, Gm^r^ | 5 × 10^6^ | ([3](#_ENREF_29)) |
| PA14.*fliI*::MAR2xT7 | *fliI* transposon mutant, Gm^r^ | 5 × 10^6^ | ([3](#_ENREF_29)) |
| PA14.*phzA2*::MAR2xT7 | *phzA2* transposon mutant, Gm^r^ | 5 × 10^6^ | ([3](#_ENREF_29)) |
| PA14.*exsA*::MAR2xT7 | *exsA* transposon mutant, Gm^r^ | 5 × 10^6^ | ([3](#_ENREF_29)) |
| PA14.*toxA*::MAR2xT7 | *toxA* transposon mutant, Gm^r^ | 5 × 10^6^ | ([3](#_ENREF_29)) |
| PA14.*lasB*::MAR2xT7 | *lasB* transposon mutant, Gm^r^ | 5 × 10^6^ | ([3](#_ENREF_29)) |
| PA14.*exoU*::MAR2xT7 | *exoU* transposon mutant, Gm^r^ | 5 × 10^6^ | ([3](#_ENREF_29)) |
| PA14.*gacA*::MAR2xT7 | *gacA* transposon mutant, Gm^r^ | 5 × 10^6^ | ([3](#_ENREF_29)) |
| PA14.*lipA*::MAR2xT7 | *lipA* transposon mutant, Gm^r^ | 5 × 10^6^ | ([3](#_ENREF_29)) |
| PA14.*wzz*::MAR2xT7 | *wzz* transposon mutant, Gm^r^ | 5 × 10^6^ | ([3](#_ENREF_29)) |
| PA14.*phoP*::MAR2xT7 | *phoP* transposon mutant, Gm^r^ | 5 × 10^6^ | ([3](#_ENREF_29)) |
| PA14.*phoQ*::MAR2xT7 | *phoQ* transposon mutant, Gm^r^ | 5 × 10^6^ | ([3](#_ENREF_29)) |
| PA14.*rhlR*::MAR2xT7 | *rhlR* transposon mutant, Gm^r^ | 5 × 10^6^ | ([3](#_ENREF_29)) |
| PA14.*lasR*::MAR2xT7 | *lasR* transposon mutant, Gm^r^ | 5 × 10^6^ | ([3](#_ENREF_29)) |
| PA14.*fleQ*::MAR2xT7 | *fleQ* transposon mutant, Gm^r^ | 5 × 10^6^ | ([3](#_ENREF_29)) |
| PA14.*rpoN*::MAR2xT7 | *rpoN* transposon mutant, Gm^r^ | 5 × 10^6^ | ([3](#_ENREF_29)) |
| ***Escherichia coli*** K-12 MG1655 | Laboratory wild type strain  F^-^ λ^+^*ilvG rfb-50 rph-1* | 5 × 10^8^ | (4) |
| ***Acinetobacter baumannii*** Ab5075 | Highly virulent, multidrug resistant clinical wound isolate | 5 × 10^8^ | (5) |
| ***Klebsiella pneumoniae*** KPLN49 | Wild type strain | 5 × 10^8^ | (6) |
| ***Enterobacter cloacae*** 218R1 | Class C chromosomal β-lactamase overproducing strain | 5 × 10^7^ | (7) |

^a^ Antibiotic resistance: Gm^r^, gentamicin.

^b^ Intradermal injection (50 μl)

^c^ References are:

1. Cheng K, Smyth RL, Govan JR, Doherty C, Winstanley C, Denning N, Heaf DP, van Saene H, Hart CA. 1996. Spread of beta-lactam-resistant *Pseudomonas aeruginosa* in a cystic fibrosis clinic. Lancet 348:639–642. <https://doi.org/10.1016/S0140-6736(96)05169-0>.

2. Rahme LG, Stevens EJ, Wolfort SF, Shao J, Tompkins RG, Ausubel FM. 1995. Common virulence factors for bacterial pathogenicity in plants and animals. Science 268:1899 –1902. <https://doi.org/10.1126/science.7604262>.

3. Liberati NT, Urbach JM, Miyata S, Lee DG, Drenkard E, Wu G, Villanueva J, Wei T, Ausubel FM. 2006. An ordered, nonredundant library of *Pseudomonas aeruginosa* strain PA14 transposon insertion mutants. Proc Natl Acad Sci U S A 103:2833–2838. <https://doi.org/10.1073/pnas.0511100103>.

4. Blattner FR, Plunkett G III, Bloch CA, Perna NT, Burland V, Riley M, Collado-Vides J, Glasner JD, Rode CK, Mayhew GF, Gregor J, Davis NW, Kirkpatrick HA, Goeden MA, Rose DJ, Mau B, Shao Y. 1997. The complete genome sequence of *Escherichia coli* K-12. Science 277:1453–1462. <https://doi.org/10.1126/science.277.5331.1453>.

5. Jacobs AC, Thompson MG, Black CC, Kessler JL, Clark LP, McQueary CN, Gancz HY, Corey BW, Moon JK, Si Y, Owen MT, Hallock JD, Kwak YI, Summers A, Li CZ, Rasko DA, Penwell WF, Honnold CL, Wise MC, Waterman PE, Lesho EP, Stewart RL, Actis LA, Palys TJ, Craft DW, Zurawski DV. 2014. AB5075, a highly virulent isolate of *Acinetobacter baumannii*, as a model strain for the evaluation of pathogenesis and antimicrobial treatments. mBio 5:e01076-14. <https://doi.org/10.1128/mBio.01076-14>.

6. Behroozian S, Svensson SL, Davies J. 2016. Kisameet clay exhibits potent antibacterial activity against the ESKAPE pathogens. mBio 7:e01842-15. <https://doi.org/10.1128/mBio.01842-15>.

7. Marchou B, Bellido F, Charnas R, Lucain C, Pechère JC. 1987. Contribution of beta-lactamase hydrolysis and outer membrane permeability to ceftriaxone resistance in *Enterobacter cloacae*. Antimicrob Agents Chemother 31:1589–1595. <https://doi.org/10.1128/AAC.31.10.1589> .
