# Supplementary material for: New Mouse Model for Chronic Infections by Gram-Negative Bacteria Enabling the Study of Anti-Infective Efficacy and Host-Microbe Interactions
Source: mBio. 2017 Feb 28;8(1):e00140-17. doi: 10.1128/mBio.00140-17 (PMC5347345; doi:10.1128/mBio.00140-17)
Supplement: TABLE S2 [file mbo001173222st2.docx]

**Table S2. Primers used in this study.**

| **Primer** | **Sequence (5' - 3')** |
| --- | --- |
| **Knockout primers** |  |
| pchA-A1 | TCGAATTCACGACAGCTTCGACGACG |
| pchA-A2 | CGAACGGATCGACGGCTTGCGTGTTCAGGTGCTGGA |
| pchD-B1 | TCCAGCACCTGAACACGCAAGCCGTCGATCCGTTCG |
| pchD-B2 | GCAAGCTTGGCGAGGAAGTCATGCGA |
| pvdG-A1 | TCGAATTCGCTTCTCCCTACGCCTTCTTCG |
| pvdG-A2 | GCCTTCTGCTCGCTGATCCGGAGACTCACTAGAACT |
| pvdL-B1 | AGTTCTAGTGAGTCTCCGGATCAGCGAGCAGAAGGC |
| pvdL-B2 | GCAAGCTTGGTCTTCGCCACGTTCGGAA |
| pchA_out1 | ACATGCAGTTGCTCGGCG |
| pchD_out2 | GCGTTGTCCATCTCCGCT |
| pvdG_out1 | AGGCCCTGCATACCGATG |
| pvdL_out2 | CCTGTGTTCTCCATGCCG |
| **qRT-PCR primers** |  |
| fliI_RT_PA14_fwd | CCTGGTGAAACGGGTCATCA |
| fliI_RT_PA14_rev | TCGATCAATGGCCTGCTCAC |
| wzz_RT_PA14_fwd | CGACAGACGTCCAAGCGTAA |
| wzz_RT_PA14_rev | CGCCAGAACAGCACCTAGAA |
| fimT_RT_PAO1_fwd | AGCTGCTGTTTGCGCTGGTT |
| fimT_RT_PAO1_rev | TCGTGCAACACACTTCGCTC |
| chpE_RT_PA14_fwd | TGTCCAACCCGAAGAACGTG |
| chpE_RT_PA14_rev | GAAACCGGCGAAGAACACCA |
| pslD_RT_PAO1_fwd | ACGCTGTACACCGTGCTCAA |
| pslD_RT_PAO1_rev | TCAGCTCGTTGGCGATTTCC |
| algR_RT_fwd | AACTCGTCTTCCAGCGCCTT |
| algR_RT_rev | CCGCTGGAAGAGGTGATCTT |
| mucC_RT_PA14_fwd | AGCATGCCGACGCACCAATA |
| mucC_RT_PA14_rev | TTTCGTGGCAGCATTGCTGG |
| rhlB_RT_PA14_fwd | ATCGAGCCGGTCTACGAGTA |
| rhlB_RT_PA14_rev | TACTTCTCGTGAGCGATGCG |
| pchB_RT_PA14_fwd | TCTGCTCGGCGATGTACC |
| pchB_RT_PA14_rev | GCTTCAAGGCCAACGAGG |
| pvdE_RT_PA14_fwd | GGCGGCATAGAACCCTTTGA |
| pvdE_RT_PA14_rev | TATCGGTCCCGATGTTCCTG |
| phzA2_RT_PA14_fwd | TTTATCCGGCCGTTCTCGAG |
| phzA2_RT_PA14_rev | CACAACGTGCGGATCTTCGA |
| lasR_RT_fwd | TCACATTGGCTTCCGAGCAG |
| lasR_RT_rev | AAACCGGTGGTTCTGACCAG |
| rhlR_RT_fwd | GTCCATGGCACCTATCCCAA |
| rhlR_RT_rev | AGACCACCATTTCCGAGGAG |
| lipA_RT_fwd | AACGGCGTGAGCTATTACTCCT |
| lipA_RT_rev | TTCTTGAAGGTCAGCGACGA |
| lipC_RT_fwd | TCCAGATGGTCCATCCGGTA |
| lipC_RT_rev | CGGCCAATGCTCCAGC |
| lasB_RT_fwd | CCAGGCCAAGAGCCTGAAG |
| lasB_RT_rev | TGTAGACCAGTTGGGCGATG |
| exsA_RT_fwd | GTTGCTGATGCTCTTCGCG |
| exsA_RT_rev | CCATGAATAGCTGCAGACGC |
| pscI_RT_PA14_fwd | GGGTCTTCATCAGGGTTTCG |
| pscI_RT_PA14_rev | ATGCAGATGCAGTGGTCGCT |
| toxA_RT_fwd | CCCGGCGAAGCATGAC |
| toxA_RT_rev | GGGAAATGCAGGCGATGA |
| gacA_RT_PA14_fwd | GATCTGGTACGCACCGGTAT |
| gacA_RT_PA14_rev | GGGCCAGTTTCAGACAGTCT |
| nirQ_RT_PA14_fwd | ACCGGCCATGAGATCGAAGT |
| nirQ_RT_PA14_rev | TACTGGACGAAGCGGGTCTT |
| relA_RT_PA14_fwd | GTGGTGACATAGCCCAGGTT |
| relA_RT_PA14_rev | TGAACTACAGCCTGCAGACC |
| 16S_RT_PA14_fwd | GGCAGGCCTAACACATGCAA |
| 16S_RT_PA14_rev | TTATCCCCCACTACCAGGCA |
